# The Influence of the Position of the Double Bond and Ring Size on the Stability of Hydrogen Bonded Complexes

**DOI:** 10.1038/s41598-017-11921-7

**Published:** 2017-09-12

**Authors:** Shumin Cheng, Shanshan Tang, Narcisse T. Tsona, Lin Du

**Affiliations:** Environment Research Institute, Shandong University, Shanda South Road 27, 250100 Shandong, China

## Abstract

To study the influence of the position of the double bond and ring size on the stability of hydrogen bonded complexes, the 1:1 complexes formed between 2,2,2-trifluoroethanol (TFE) and three heterocyclic compounds including 2,3-dihydrofuran (2,3-DHF), 2,5-dihydrofuran (2,5-DHF) and 3,4-dihydropyran (3,4-DHP) were investigated systematically. The formation of hydrogen bonded TFE−2,3-DHF, TFE−2,5-DHF and TFE−3,4-DHP complexes were identified by gas phase FTIR spectroscopy at room temperature, and the OH-stretching fundamental transition of TFE was red shifted upon complexation. The competition between the O atom and π-electrons bonding sites within the complexes was studied, and the O−H···π type hydrogen bond was found to be less stable than the O−H···O in all three cases. The observed red shifts of the OH-stretching fundamental transitions in the complexes were attributed to the formation of O−H···O hydrogen bond. Equilibrium constants of the complexation reactions were determined from measured and calculated OH-stretching fundamental intensities. Both theoretical calculations and experimental results reveal that the hydrogen bond strengths in the complexes follow the sequence: TFE−2,5-DHF > TFE−2,3-DHF ≈ TFE−3,4-DHP, thus the position of the double bond exerts significantly larger influence than ring size on the stability of the selected hydrogen bonded complexes.

## Introduction

Atmospheric nanometer sized clusters play an important role in the formation and growth of aerosol particles. These clusters are usually stabilized by hydrogen bonding interactions^1–5^. Investigation of hydrogen bonded complexes formed between atmospheric relevant molecules is important in understanding the characteristics of particles at the molecular level. According to previous researches, hydrogen bonded complexes can be formed among a number of molecules, such as alcohols, amines and aromatic compounds^5–8^. Alcohols have the ability to form hydrogen bonded complexes either as hydrogen bond donor or hydrogen bond acceptor through the H atom or the O atom of the hydroxyl group, respectively. A particular case of atmospherically relevant alcohol in complex formation is the 2,2,2-trifluoroethanol (TFE), one of the widely used fluorinated alcohols that is available on a large commercial scale. It has been used in many reactions as solvent, cosolvent, catalyst, and it is also important for the preparation of many pharmaceuticals and other chemicals^9–12^. Increased production and use of TFE will lead to an increase of its emission into the atmosphere. The electron withdrawing CF_3_ group present in TFE enhances its acidity and makes it a strong proton donor^13, 14^. Considering the close correlation between hydrogen bonded clusters and new particles formation, it is necessary to study the interactions of TFE with compounds present in the atmosphere.

Hydrogen bonding interactions between TFE and various atmospheric components have been explored by many studies, both theoretically and experimentally. Hydrogen bonded complexes of TFE with ammonia, tetrahydrofuran or diethyl ether in the gas phase studied with Fourier transform infrared (FTIR) spectrometer have been reported, and the formation of these complexes were identified by observed red shifts of the OH-stretching fundamental transitions^9, 15^. Using alcohols including TFE, ethanol (EtOH), and methanol (MeOH) as hydrogen bond donors, and dimethylamine (DMA) or trimethylamine (TMA) as acceptor, the strengths of the O−H···N hydrogen bond in the alcohol–amine complexes were examined using vapor phase FTIR spectroscopy and quantum chemical calculations. Based on the observed red shifts and calculated results, TFE–amine complexes were found to be significantly more stable than EtOH/MeOH–amine complexes^16^. In a similar study, using same hydrogen bond donors but the trimethylphosphine (TMP) as acceptor, the TFE–TMP complex was found to be most stable among the TFE/EtOH/MeOH–TMP complexes^17^, indicating that the presence of the CF_3_ group facilitates the formation of strong hydrogen bond. Hydrogen bonding interactions between TFE and dimethylether or dimethylsulfide have been investigated with FTIR and density functional theory (DFT) calculations, by which the strengths of O−H···O and O−H···S hydrogen bonds were found to be very similar^18^. Using similar methods, another study of complexes formed between TFE and ethylene oxide or ethylene sulfide also identified that O−H···O and O−H···S hydrogen bonds have comparable strength^19^.

Dihydrofurans have been found to be produced from many sources. Natural source from biomass burning has been reported widely^20, 21^, and biomass burning emission of 2,3-dihydrofuran (2,3-DHF) is included in the emission inventory of atmospheric-chemistry transport models aimed at the emissions of trace gases and aerosols^22^. There are also anthropogenic sources including agricultural processes and food processing^23^. Furthermore, dihydrofurans present in the atmosphere are both primary and secondary pollutants. Different dihydrofurans from alkanes photooxidation, which are involved in the formation of secondary organic aerosol have also been found^24–26^. When investigating the oxidation of 2,3-DHF, 2,5-dihydrofuran (2,5-DHF) and 3,4-dihydropyran (3,4-DHP) in the daytime with free radicals, the influence of the position of the double bond and ring size were discussed in the comparison of their rate coefficients^27^. The rate coefficients of 2,3-DHF and 3,4-DHP were found to be almost twice higher than that of 2,5-DHF when reacting with the OH radical, and the difference in reactivity was attributed to the conjugation of the double bond with the lone pair of O atom. Thus, we can speculate that the hydrogen bonding interactions between TFE and these heterocyclic molecules will also be influenced by the two factors. On the other hand, with the electron-rich O atom and π-electrons in 2,3-DHF, 2,5-DHF and 3,4-DHP, there are two bonding sites available in each proton acceptor, which facilitate the formation of both O−H···O and O−H···π hydrogen bonds with TFE, and there is a possibility of competition between the two bonding sites^8, 23, 28–30^.

From the definition recommended by IUPAC, in a X−H···Y−Z hydrogen bond where X−H stands for hydrogen bond donor and Y−Z represents the acceptor, X should be more electronegative than H, but no specific limitation is put on the acceptor except that it should be an electron rich zone, such as O, N, F and π-electrons^31^. FTIR spectroscopy is a primary and helpful technique to study hydrogen bonding interactions^3^, because the red shift and intensity enhancement of vibration in X−H upon hydrogen bond formation can be directly obtained from infrared spectra. These parameters are important in identifying and characterizing hydrogen bond^7^. For the past few years, both matrix isolation FTIR and gas phase FTIR spectroscopies have been widely used in the investigation of complexation between molecules. The hydrogen bonding interactions between benzene and a series of fluorophenol were investigated using matrix isolation FTIR spectroscopy in argon matrix. The formation of O−H···π hydrogen bonded fluorophenol−benzene complexes was identified by the observed frequency shifts in infrared spectra, and the results were further confirmed by quantum chemical studies^32^. The hydrogen bonding interaction between DMA and TMP was detected with FTIR spectroscopy in the gas phase. The feature of the NH-stretching fundamental transition, found to appear at 3350 cm^−1^ in the infrared spectra, indicated a red shift of 24 cm^−1^ relative to that of DMA monomer, and in this case, the formation of N−H···P hydrogen bonded DMA−TMP complex was identified^6^. Compared with matrix isolation FTIR, gas phase FTIR spectroscopy is a better choice in the study of atmospheric relevant molecules, since the experimental condition of the latter is much closer to the atmospheric environment and the interference of the matrix is avoided. Meanwhile, the system is in equilibrium thus enabling to study the thermodynamic stability of the hydrogen bonded complexes^17, 33, 34^.

In the present work, we investigated the hydrogen bonding interactions between TFE and three heterocyclic compounds (2,3-DHF, 2,5-DHF and 3,4-DHP) systematically. The differences between properties of TFE−2,3-DHF and TFE−2,5-DHF complexes allow us to investigate the influence of the position of the double bond on the hydrogen bond formation, while the effect of ring size can be evaluated from the differences between properties of TFE−2,3-DHF and TFE−3,4-DHP complexes. The infrared spectra of samples were investigated by gas phase FTIR spectroscopy at room temperature. In addition, theoretical calculations with DFT methods were employed to study the structural, vibrational and thermodynamic properties of the TFE−complexes. There are three critical goals in this study: (1) to investigate the characteristics of the hydrogen bonded complexes formed between TFE and three heterocyclic compounds; (2) to elucidate the influence of the position of the double bond and ring size on the formation of hydrogen bonded complexes; (3) to study the competition between the O atom and π-electrons bonding sites of the selected heterocyclic molecules in hydrogen bonding interactions.

## Results and Discussion

### Optimized geometries

**Figure 1:**
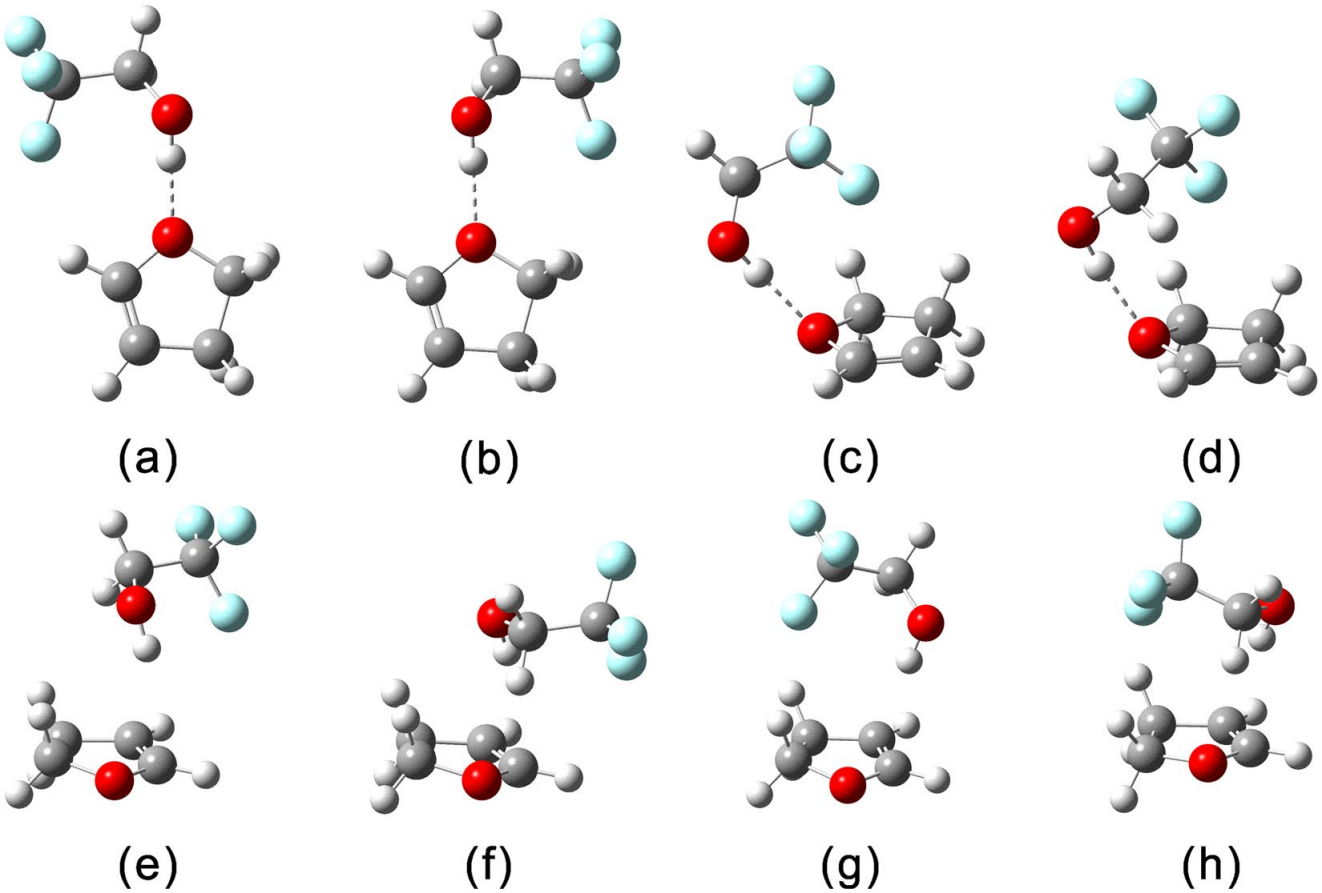
Optimized structures of the TFE−2,3-DHF complex calculated at the B3LYP-D3/aug-cc-pVTZ level of theory.

The optimized structures of hydrogen bonded TFE−2,3-DHF, TFE−2,5-DHF and TFE−3,4-DHP complexes at the B3LYP-D3/aug-cc-pVTZ level of theory are shown in Figs. 1 and S1. Two minimum energy conformers were found for the TFE monomer, *trans* and *gauche*. The structural difference between these two conformers is the dihedral angle of CCOH. It was previously demonstrated from a computational study that TFE *gauche-*conformer is more stable than the *trans-*conformer, with an energy difference of 8 kJ mol^−1^ at the MP2/aug-cc-pVTZ level of theory^35^. Experimental studies of bare TFE with vapor phase FTIR also found the dominance of the *gauche-*conformer over the *trans-*conformer^9, 36^. As seen from Figs. 1 and S1, only *gauche-*conformer was found in optimized structures of the complexes in the present study, which is in agreement with former studies^13, 18, 19^. However, there is still controversy about the reason why the *gauche-*conformer is more stable. Many studies suggested that the stability of the *gauche-*conformer is due to the formation of intramolecular O−H···F hydrogen bond^15, 37–39^, while there is also an interpretation that the dominance of the *gauche-*conformer originates from a decrease of repulsion force between the oxygen electronic pair and the fluorine atom clouds compared to the *trans-*conformer^40^.

Eight isomers were predicted for the TFE−2,3-DHF complex, labeled from (a) to (h), including four O−H···O and four O−H···π type isomers, with TFE acting as a proton donor to the O atom and π-electrons of 2,3-DHF. For the TFE−2,3-DHF (a) and TFE−2,3-DHF (b), TFE is in the same plane as the 2,3-DHF ring, and the difference between them is the relative position between the CF_3_ group of TFE and the C=C bond of 2,3-DHF ring. Two more complex structures of O−H···O type were explored, where TFE approaches the O atom of 2,3-DHF from above the plane of the ring, then TFE−2,3-DHF (c) and TFE−2,3-DHF (d) were found to be minimum energy structures. When the OH bond is vertically above the 2,3-DHF ring and pointing to the π-electrons, like the structures of the O−H···π hydrogen bonded complex formed between trifluoroacetic acid and benzene^41^, four minimum energy geometries were got, TFE−2,3-DHF (e) and TFE−2,3-DHF (f) with CF_3_ group pointing away from 2,3-DHF, TFE−2,3-DHF (g) and TFE−2,3-DHF (h) with CF_3_ group directly above 2,3-DHF ring. Similar structures were obtained with other DFT methods used here, except conformers TFE−2,3-DHF (c) and TFE−2,3-DHF (d) when using B3LYP method. With respect to complexation between TFE and 3,4-DHP, similar geometries with TFE−2,3-DHF were predicted by the four functionals employed, the structures at the B3LYP-D3/aug-cc-pVTZ level are shown in Fig. S1. Again, conformers TFE−3,4-DHP (c) and TFE−3,4-DHP (d) were not predicted as minimum energy structures at the B3LYP/aug-cc-pVTZ level of theory. Thus, the structure of TFE−3,4-DHP is comparable to that of TFE−2,3-DHF. Due to the symmetry of 2,5-DHF, four isomers were identified for TFE−2,5-DHF as shown in Fig. S1, and no minimum energy structures were observed for conformers TFE−2,5-DHF (b) and TFE−2,5-DHF (c) at the B3LYP/aug-cc-pVTZ level of theory. Optimized structures of the TFE−2,5-DHF complex are similar to those obtained in a former investigation of the complexation between MeOH and 2,5-dimethylfuran (2,5-DMFu) at the B3LYP-D3/aVTZ level of theory, where three conformers were obtained, among which, two O−H···O conformers with the methyl moiety of MeOH molecule located above or pointing away from the π system of 2,5-DMFu, and one O−H···π conformer with MeOH molecule positioned above 2,5-DMFu^42^. For the TFE−2,5-DHF complex, the O−H···π type TFE−2,5-DHF (c) conformer with CF_3_ group pointing away from 2,5-DHF is also a minimum energy structure.

A summary of the structure parameters of the complexes at the B3LYP-D3/aug-cc-pVTZ level of theory is given in Table 1. Results obtained at other levels of theory are given in Table S1 in the supplementary material. Geometric parameters such as the OH bond length (*r*_(OH)_) and the change in the OH bond length upon complexation (Δ*r*_(OH)_) for all the isomers of the complexes are listed in Table 1. For the O−H···O type isomers of the complexes, the intermolecular hydrogen bond distance (*r*_(HB)_) and intermolecular hydrogen bond angle (*θ*_(HB)_) are also given. The positive values of Δ*r*_(OH)_ indicate that the OH bond is lengthened after the formation of hydrogen bond. In general, the elongation in the OH bond lengths of 0.0150 Å in O−H···O type TFE−2,5-DHF isomers is longer than those of same type TFE−2,3-DHF and TFE−3,4-DHP isomers, ranging from 0.0101 to 0.0111 Å and from 0.0096 to 0.0106 Å, respectively. However, for O−H···π type isomers, the trend is exactly opposite, with the elongations in the OH bond lengths in TFE−2,5-DHF isomers being 0.0053 and 0.0056 Å, which are generally shorter than those of TFE−2,3-DHF and TFE−3,4-DHP isomers that range from 0.0048 to 0.0082 Å and 0.0062 to 0.0084 Å, respectively. Meanwhile, it can be seen that the elongation values of all the isomers of TFE−2,3-DHF and TFE−3,4-DHP are similar with each other, with differences less than 0.0014 Å. In an ideal hydrogen bonded complex, the hydrogen bond angle is close to linear (180°)^34^. All the O−H···O isomers of the three complexes shown in Table 1 exhibit bond angles very closed to 180°, with a smallest deviation of 1.0° and a largest deviation of 12.2° belonging to TFE−2,5-DHF (a) and TFE−3,4-DHP (d), respectively. Furthermore, the intermolecular hydrogen bond distances in isomers of TFE−2,5-DHF exhibit values about 0.05 Å smaller than those of TFE−2,3-DHF and TFE−3,4-DHP, which have almost same values. According to criteria of hydrogen bond introduced by IUPAC, the formation of stronger hydrogen bond including features of greater lengthening of X−H bond, shorter H···Y distance and closer to linear X−H···Y angle^31, 43^, indicating that the strengths of the TFE−2,3-DHF and TFE−3,4-DHP complexes are almost equivalent, based on their structure parameters. Considering the discrepancy on the elongation in O−H···O and O−H···π type isomers of TFE−2,5-DHF relative to those of the TFE−2,3-DHF and TFE−3,4-DHP complexes, more evidence is needed to give the order of strength including TFE−2,5-DHF complex.

**Table 1:** Selected optimized geometric parameters of the complexes at the B3LYP-D3/aug-cc-pVTZ level (angles in degrees and bond lengths in Å).

Conformer	Type of H-bond	*r*_(OH)_^a^	Δ*r*_(OH)_^b^	*r*_(HB)_^c^	*θ*_(HB)_^d^
**TFE−2,3-DHF**					
(a)	O−H···O	0.9738	0.0108	1.8148	176.9
(b)	O−H···O	0.9739	0.0108	1.8137	178.1
(c)	O−H···O	0.9742	0.0111	1.8365	174.2
(d)	O−H···O	0.9731	0.0101	1.8418	171.2
(e)	O−H···π	0.9712	0.0082		
(f)	O−H···π	0.9691	0.0060		
(g)	O−H···π	0.9708	0.0078		
(h)	O−H···π	0.9678	0.0048		
**TFE−2,5-DHF**					
(a)	O−H···O	0.9780	0.0150	1.7597	179.0
(b)	O−H···O	0.9780	0.0150	1.7787	173.0
(c)	O−H···π	0.9686	0.0056		
(d)	O−H···π	0.9684	0.0053		
**TFE−3,4-DHP**					
(a)	O−H···O	0.9735	0.0105	1.8139	177.2
(b)	O−H···O	0.9737	0.0106	1.8104	172.7
(c)	O−H···O	0.9736	0.0106	1.8316	173.7
(d)	O−H···O	0.9727	0.0096	1.8309	167.8
(e)	O−H···π	0.9714	0.0084		
(f)	O−H···π	0.9700	0.0069		
(g)	O−H···π	0.9706	0.0076		
(h)	O−H···π	0.9692	0.0062		

^a^OH bond length. ^b^Δ*r*_(OH)_ = *r*_complex_ − *r*_TFE_, is the change in the OH bond length upon complexation. ^c^Intermolecular hydrogen bond distance. ^d^Intermolecular hydrogen bond angle, i.e., *θ*_(O−H_···_O)_.

The elongations in the OH bond lengths in O−H···π type isomers are much shorter than O−H···O type in all three cases (Table 1), indicating that the O−H···O type isomers are more stable than the corresponding O−H···π type isomers. It confirms that a competition exists between the O atom and π-electrons bonding sites, and the docking preference of TFE to the former bonding site than the latter one is obvious. However, the N−H···π hydrogen bond was found to be more stable than the conventional N−H···O hydrogen bond in a jet-cooled study of indole–furan heterodimer^44^. This difference is due to the deficiency of π-electrons in dihydrofuran compared with furan. When the MeOH binding to diphenyl ether, the aromatic π system winning over the ether oxygen was shown by the employment of multi-experimental approach and DFT calculations^8^. When MeOH docks onto 2,3-benzofuran, 1:1 hydrogen bonded O−H···O and O−H···π clusters were found with comparable strength using theoretical calculations, which was further evidenced along with fluorescence-detected infrared spectroscopy and dispersed fluorescence spectroscopy^28^. In a docking preference study of MeOH onto anisole using supersonic jet-FTIR, carried out systematically by ring methylation of anisole, the subtle balance between O−H···O and O−H···π structures was found to vary by one order of magnitude through single to triple methylation of the aromatic ring and introduction of a single tert-butyl substituent^30^.

### Experimental and calculated OH-stretching fundamental transitions

**Figure 2:**
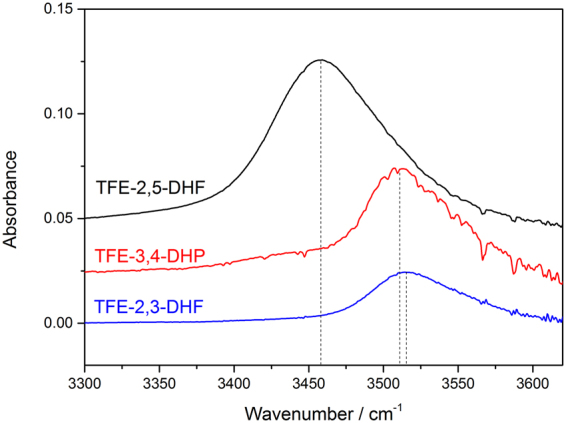
Spectra of the complexes *in the OH﻿-stretching band region;*_OH_. The spectra were recorded with a 20 cm path length cell and with pressure: 1176 Pa TFE + 4732 Pa 2,5-DHF, 3657 Pa TFE + 4926 Pa 3,4-DHP, 1258 Pa TFE + 5570 Pa 2,3-DHF. The spectra have been offset.

The hydrogen bonded TFE−complexes using FTIR spectrometer were detected in the gas phase at room temperature. Spectra of the complexes are summarized in Fig. 2 for direct comparison. The experimental infrared spectra of monomers and corresponding mixtures of TFE with 2,3-DHF, 2,5-DHF and 3,4-DHP with background subtracted in the region of the OH-stretching fundamental transitions are presented in Fig. S2(a–c), respectively. To confirm reproducibility and make sure that the complexes formed are binary, FTIR spectroscopic experiments were performed for five times at different combinations of pressure for each complex. In the spectra of TFE monomer, the OH-stretching vibration modes are very strong and present in the region of 3500–3700 cm^−1^ ^45^. The peak observed at 3657 cm^−1^ can be assigned to the OH-stretching fundamental transition, which is consistent with earlier gas phase observations^15, 17, 19^. The formation of hydrogen bond usually results in the elongation of the OH bond which further induces the red shift of the OH-stretching fundamental band in infrared spectra^46^. In Fig. S2(a), compared to the spectra of TFE and 2,5-DHF monomers, a new band appears in the spectra of the mixture at a frequency lower than 3657 cm^−1^, and the intensity of the new band was found to increase with the pressure increase of monomers. In this case, the appearance of the new band arising from the formation of the TFE−2,5-DHF complex can be confirmed. Similar results can be found in Fig. S2(b) and (c). Spectra of complexes were obtained by subtracting spectra of monomers from those of the mixtures. In the spectral subtraction, a scaling factor was applied to the pure TFE spectra so that the TFE transitions matched in regions where only TFE was absorbed. Spectra of the heterocyclic compounds were also calibrated in this way. By doing this, the contribution of the monomer spectra could be completely removed from the spectra of their mixture, and the residue spectra was from the complex. The interference of trace amounts of water vapor present as the “spiky” peaks at around 3600 cm^−1^, could not be completely removed from spectra subtraction, but had little effect on the spectra of complexes.

After the spectral subtraction of spectra recorded for the samples, the features at 3515, 3458 and 3510 cm^−1^ could be assigned to the OH-stretching fundamental of the TFE−2,3-DHF, TFE−2,5-DHF and TFE−3,4-DHP complexes, respectively. The assignment of the features can be confirmed by the deconvolution fittings of the OH-stretching fundamental transition bands with Lorentzian functions. These deconvolution fittings of complexes spectra are given in Fig. S3. Considering the OH-stretching fundamental transition of TFE monomer positioned at 3657 cm^−1^, the red shifts in the OH-stretching fundamental due to complexation of TFE with 2,3-DHF, 2,5-DHF and 3,4-DHP are 142, 199 and 147 cm^−1^, accordingly. The observed red shift of 199 cm^−1^ in TFE−2,5-DHF complex is larger than that of the MeOH−2,5-DHF complex found in the range of 103–178 cm^−1^ ^23^, which can be attributed to the electron-withdrawing CF_3_ group. From Fig. 2, we can see that the red shifts in the spectra of TFE−2,3-DHF and TFE−2,5-DHF show a significant difference of 57 cm^−1^, which implies that the position of the double bond has a great impact on the position of the OH-stretching fundamental transition in the gas phase. It is also notable that the red shifts in TFE−2,3-DHF and TFE−3,4-DHP are of similar extent, with a difference of only 5 cm^−1^, thus the change of ring size has a much smaller impact.

The calculated OH-stretching harmonic frequencies and intensities of TFE monomer and TFE−complexes at the B3LYP-D3/aug-cc-pVTZ level of theory are listed in Table 2. Results obtained at other levels of theory are given in Table S2 in the supplementary material. The calculations were based on optimized structures of the complexes. The red shifts and intensity enhancements (*f*/*f*_*TFE*_) are also included, which can be considered as criteria for the strength of a hydrogen bond^47, 48^. It can be seen that the red shifts of O−H···O type TFE−2,5-DHF isomers varying from 311 to 314 cm^−1^ represents isomers with higher stability, followed by those of TFE−2,3-DHF varying from 215 to 234 cm^−1^, and those of TFE−3,4-DHP varying from 204 to 223 cm^−1^, respectively. The similar values of the latter two represent isomers with comparable strength. Observed OH-stretching fundamental frequencies and red shifts are also given for comparison. For the studied complexes, all the calculated frequency shifts of O−H···O type isomers are larger than observed values. Meanwhile, calculated OH-stretching fundamental transitions of O−H···O type isomers for the complexes are significantly closer to observed values compared to O−H···π type isomers, and among the four functionals used, results from B3LYP-D3 accord best with observed values. With larger red shifts, isomers of the complexes with O−H···O hydrogen bonds are more stable, indicating that the O atom is a much stronger bonding site than π-electrons. Thus, the experimental products can be safely assigned to O−H···O bonded complexes and the red shifts of the complexes relative to TFE monomer should be attributed to the formation of O−H···O hydrogen bond. Since the red shifts of the O−H···O type isomers shown in Table 2 are very close to each other for individual complexes, the O−H···O type isomers may coexist in the gas phase. The calculated frequencies are in good agreement with the experimental results in general. Similar trend of red shifts in O−H···O type complexes can be found in both observed and calculated data sets, having the sequence of TFE−2,5-DHF > TFE−2,3-DHF ≈ TFE−3,4-DHP. In addition, calculated OH-stretching intensities of the complexes are enhanced relative to that of TFE monomer due to hydrogen bonding interactions, with enhancement values of the O−H···O type isomers of the complexes showing similar trends with red shifts. Then the order of hydrogen bond strengths for the TFE−complexes including TFE−2,5-DHF follows the sequence shown above.

**Table 2:** Calculated OH-stretching wavenumbers and oscillator strengths of TFE and complexes at the B3LYP-D3/aug-cc-pVTZ level.

Conformer	Type of H-bond	$\tilde{{\boldsymbol{v}}}/{\bf{c}}{{\bf{m}}}^{{\boldsymbol{-}}{\bf{1}}}$		${\boldsymbol{\Delta }}\tilde{{\boldsymbol{v}}}/{\bf{c}}{{\bf{m}}}^{{\boldsymbol{-}}{\bf{1}}{\rm{a}}}$		*f*	*f*/*f*_*TFE*_
		Calculated	Observed	Calculated	Observed		
TFE		3804	3657			9.1 × 10^−6^	
**TFE−2,3-DHF**							
(a)	O−H···O	3576	3515	228	142	1.8 × 10^−4^	19.4
(b)	O−H···O	3574		229		1.8 × 10^−4^	19.2
(c)	O−H···O	3570		234		1.5 × 10^−4^	16.1
(d)	O−H···O	3588		215		1.3 × 10^−4^	13.8
(e)	O−H···π	3619		184		9.5 × 10^−5^	10.4
(f)	O−H···π	3664		140		6.1 × 10^−5^	6.7
(g)	O−H···π	3625		179		1.1 × 10^−4^	12.1
(h)	O−H···π	3684		119		6.8 × 10^−5^	7.5
**TFE−2,5-DHF**							
(a)	O−H···O	3490	3458	314	199	1.9 × 10^−4^	20.9
(b)	O−H···O	3493		311		1.6 × 10^−4^	17.7
(c)	O−H···π	3672		132		8.8 × 10^−5^	9.7
(d)	O−H···π	3676		127		8.3 × 10^−5^	9.1
**TFE−3,4-DHP**							
(a)	O−H···O	3581	3510	223	147	1.8 × 10^−4^	19.4
(b)	O−H···O	3580		223		1.7 × 10^−4^	19.1
(c)	O−H···O	3582		222		1.4 × 10^−4^	15.5
(d)	O−H···O	3600		204		1.3 × 10^−4^	14.3
(e)	O−H···π	3613		190		9.7 × 10^−5^	10.6
(f)	O−H···π	3643		161		7.0 × 10^−5^	7.7
(g)	O−H···π	3627		177		1.1 × 10^−4^	12.1
(h)	O−H···π	3655		148		8.4 × 10^−5^	9.2

^a^ $${\rm{\Delta }}{\tilde{v}}_{{\rm{OH}}}={\tilde{v}}_{{\rm{TFE}}}-{\tilde{v}}_{{\rm{complex}}}$$.

### Interaction energies

The binding energies (*BE*s) together with the thermodynamic parameters including enthalpy of formation (${\rm{\Delta }}{H}_{calc}^{\theta }$), Gibbs free energy of formation (${\rm{\Delta }}{G}_{calc}^{\theta }$) and equilibrium constants (${K}_{eq}^{calc}$) of the complexes at 298 K, calculated at the B3LYP-D3/aug-cc-pVTZ level of theory are listed in Table 3. Results obtained at other levels of theory are given in Table S3. It can be seen that effects of the zero-point vibrational energy (ZPVE) on *BE*s are up to 2.6–5.1 kJ mol^−1^, while the basis set superposition error (BSSE) values vary from 0.9 to 1.3 kJ mol^−1^ for the relatively large basis set used here.

**Table 3:** Calculated binding energy (*BE*), enthalpy of formation (${\rm{\Delta }}{H}_{calc}^{\theta }$), Gibbs free energy of formation (${\rm{\Delta }}{G}_{calc}^{\theta }$) and equilibrium constant (${K}_{eq}^{calc}$) at 298 K for the complexes at the B3LYP-D3/aug-cc-pVTZ level^a^.

Conformer	Type of H-bond	*BE* ^b^	ZPVE	BSSE	${\boldsymbol{\Delta }}{{\boldsymbol{H}}}_{{\boldsymbol{calc}}}^{{\boldsymbol{\theta }}}$	${\boldsymbol{\Delta }}{{\boldsymbol{G}}}_{{\boldsymbol{calc}}}^{{\boldsymbol{\theta }}}$	${{\boldsymbol{K}}}_{{\boldsymbol{eq}}}^{{\boldsymbol{calc}}}$
**TFE−2,3-DHF**							
(a)	O−H···O	−28.4	4.4	1.0	−27.4	8.3	3.6 × 10^−2^
(b)	O−H···O	−28.7	4.2	1.0	−27.6	7.1	5.7 × 10^−2^
(c)	O−H···O	−27.5	4.6	1.2	−27.0	11.8	8.6 × 10^−3^
(d)	O−H···O	−29.7	5.1	1.1	−29.3	10.6	1.4 × 10^−2^
(e)	O−H···π	−19.9	3.3	0.9	−18.4	16.4	1.3 × 10^−3^
(f)	O−H···π	−23.6	3.9	1.0	−22.4	15.8	1.7 × 10^−3^
(g)	O−H···π	−20.6	3.7	1.1	−19.6	17.6	8.1 × 10^−4^
(h)	O−H···π	−23.8	3.8	1.2	−22.8	15.4	2.0 × 10^−3^
**TFE−2,5-DHF**							
(a)	O−H···O	−33.7	5.1	1.0	−32.9	3.9	2.1 × 10^−1^
(b)	O−H···O	−33.2	5.3	1.3	−33.0	6.7	6.6 × 10^−2^
(c)	O−H···π	−16.3	2.6	0.9	−14.4	16.1	1.5 × 10^−3^
(d)	O−H···π	−16.5	2.8	1.0	−14.9	18.7	5.4 × 10^−4^
**TFE−3,4-DHP**							
(a)	O−H···O	−28.8	4.2	1.0	−27.6	6.5	7.2 × 10^−2^
(b)	O−H···O	−28.6	4.2	1.0	−27.3	7.5	4.9 × 10^−2^
(c)	O−H···O	−26.9	4.2	1.2	−26.0	10.2	1.6 × 10^−2^
(d)	O−H···O	−30.5	4.5	1.2	−29.6	8.1	3.9 × 10^−2^
(e)	O−H···π	−21.5	3.4	1.1	−20.1	15.4	2.0 × 10^−3^
(f)	O−H···π	−24.7	4.0	1.0	−23.5	15.8	1.7 × 10^−3^
(g)	O−H···π	−22.2	3.4	1.2	−21.0	16.0	1.6 × 10^−3^
(h)	O−H···π	−24.1	3.8	1.3	−23.1	16.1	1.5 × 10^−3^

^a^Energies are in kJ mol^−1^. ^b^ *BE* corrected with ZPVE and BSSE.

The *BE* of a complex is an important criterion to measure the strength of a hydrogen bond. In the selected heterocyclic molecules, conformations of the complexes with higher interaction energy correspond to the OH group of TFE pointing directly to the O atom of the heterocyclic molecules, while the π-electrons represents a much weaker bonding site. This is evident from the former conclusion that the TFE−complexes are stabilized by O−H···O hydrogen bonds in the gas phase. In addition, the similarity in *BE*s of O−H···O type isomers for individual complexes supports the speculation that O−H···O type isomers coexist in the gas phase. Meanwhile, the *BE*s of O−H···O type TFE−2,5-DHF isomers are evidently more negative compared to corresponding same type TFE−2,3-DHF isomers, while the values of TFE−2,3-DHF and TFE−3,4-DHP are very close to each other despite changing from five-membered ring to six-membered ring, which implies that the position of the double bond has an important impact on complexation while the change of ring size has little to no effect. Predictions of *BE*s with other functionals in Table S3 show the same trend, and it can be observed that the values for isomers of the TFE−complexes vary with different functionals employed. Differences in *BE*s from M06-2X, ωB97X-D and B3LYP-D3 are within 3 kJ mol^−1^, while *BE*s calculated with the B3LYP functional are significantly underestimated. Thus, *BE*s of the selected complexes are sensitive to the choice of DFT methods.

The ${\rm{\Delta }}{G}_{calc}^{\theta }$ values listed in Table 3 are all positive, and become less positive as the *BE*s increase. Hydrogen bonded MeOH−TMA and MeOH−DMA complexes were detected with gas phase FTIR spectroscopy^48^, and the ${\rm{\Delta }}{G}_{calc}^{\theta }$ values calculated for the complexes were 11.4 and 10.6 kJ mol^−1^ at the B3LYP/aug-cc-pVTZ level of theory, corresponding to *BE*s of −19.7 and −20.8 kJ mol^−1^, respectively. The formation of hydrogen bonded TFE−dimethylether and TFE−dimethylsulfide were identified in the gas phase with FTIR spectroscopy^18^, the ${\rm{\Delta }}{G}_{calc}^{\theta }$ values of the complexes were found to be 4.2 and 8.1 kJ mol^−1^ at the B3LYP-D3/aug-cc-pVTZ level of theory, and the *BE*s for the complexes were −31.3 and −28.1 kJ mol^−1^, accordingly. The possibility of complexation between two molecules is relevant to ${\rm{\Delta }}{G}_{calc}^{\theta }$. It can be seen from Table 3 that smaller ${\rm{\Delta }}{G}_{calc}^{\theta }$ values of O−H···O type TFE−2,5-DHF isomers suggest higher possibility of hydrogen bond formation than same type TFE−2,3-DHF/3,4-DHP isomers. At thermal equilibrium, the relationship between ${\rm{\Delta }}{G}_{calc}^{\theta }$ and ${K}_{eq}^{calc}$ can be expressed by the following equation:

1 $${\rm{\Delta }}{G}_{calc}^{\theta }=-RT\,\mathrm{ln}({K}_{eq}^{calc})$$

where R is the gas constant and T is the absolute temperature. From equation (1), the calculated values of ${K}_{eq}^{calc}$ can be obtained, which are listed in Tables 3 and S3. Equilibrium constant can also be determined from measured and calculated OH-stretching fundamental intensities, which can be considered as a reference for the ${K}_{eq}^{calc}$ obtained through DFT methods.

### Determination of the equilibrium constant

Equilibrium constants of the complexation reactions provide a deep insight into the thermostability of the TFE−complexes in the gas phase at room temperature. When the hydrogen bonded complexes and corresponding monomers in the gas cell are in equilibrium state, the equilibrium constant *K*_*eq*_ can be determined by the following equation:

2 $${K}_{eq}=\frac{{p}_{complex}/{p}^{\theta }}{{p}_{TFE}/{p}^{\theta }\times {p}_{2,5 \mbox{-} DHF/2,3 \mbox{-} DHF/3,4 \mbox{-} DHP}/{p}^{\theta }}$$

In equation (2), *p* *θ* is the standard pressure (1 bar = 10^5^ Pa), *p*_*TFE*_ and *p*_*2,5-DHF/2,3-DHF/3,4-DHP*_ are the pressures of the monomers in Pa, which are determined from pressures of monomers measured with pressure gauge multiplied by corresponding scaling factors used for spectral subtraction. However, the amount of the complex formed is so small that its pressure (*p*_*complex*_) cannot be detected directly. The quantification of the complex is based on observed integrated absorbance and calculated vibrational intensity of the OH-stretching fundamental transition, using the formula below:

3 $${p}_{complex}=3.5910\times {10}^{-7}({K}^{-1}\,Pa\,m\,cm)\frac{T\times \int A(\tilde{\nu })d\tilde{\nu }}{{f}_{calc}\times l}$$

where *T* is the experimental temperature in K, *A* is the observed absorbance, $\int A(\tilde{\nu })d\tilde{\nu }$ is the observed integrated absorbance in cm^−1^, *l* is the optical path length in m and *f*_*calc*_ is the vibrational intensity calculated at the B3LYP-D3/aug-cc-pVTZ level of theory. The *f*_*calc*_ used in this work are 1.3 × 10^−4^, 1.9 × 10^−4^, and 1.3 × 10^−4^ for TFE−2,3-DHF, TFE−2,5-DHF and TFE−3,4-DHP, respectively, corresponding to *f*_*calc*_ of conformers with most negative *BE*s for every complex. This formula has been successfully used to determine pressures of complexes in previous studies^16, 17, 33, 48^.

**Figure 3:**
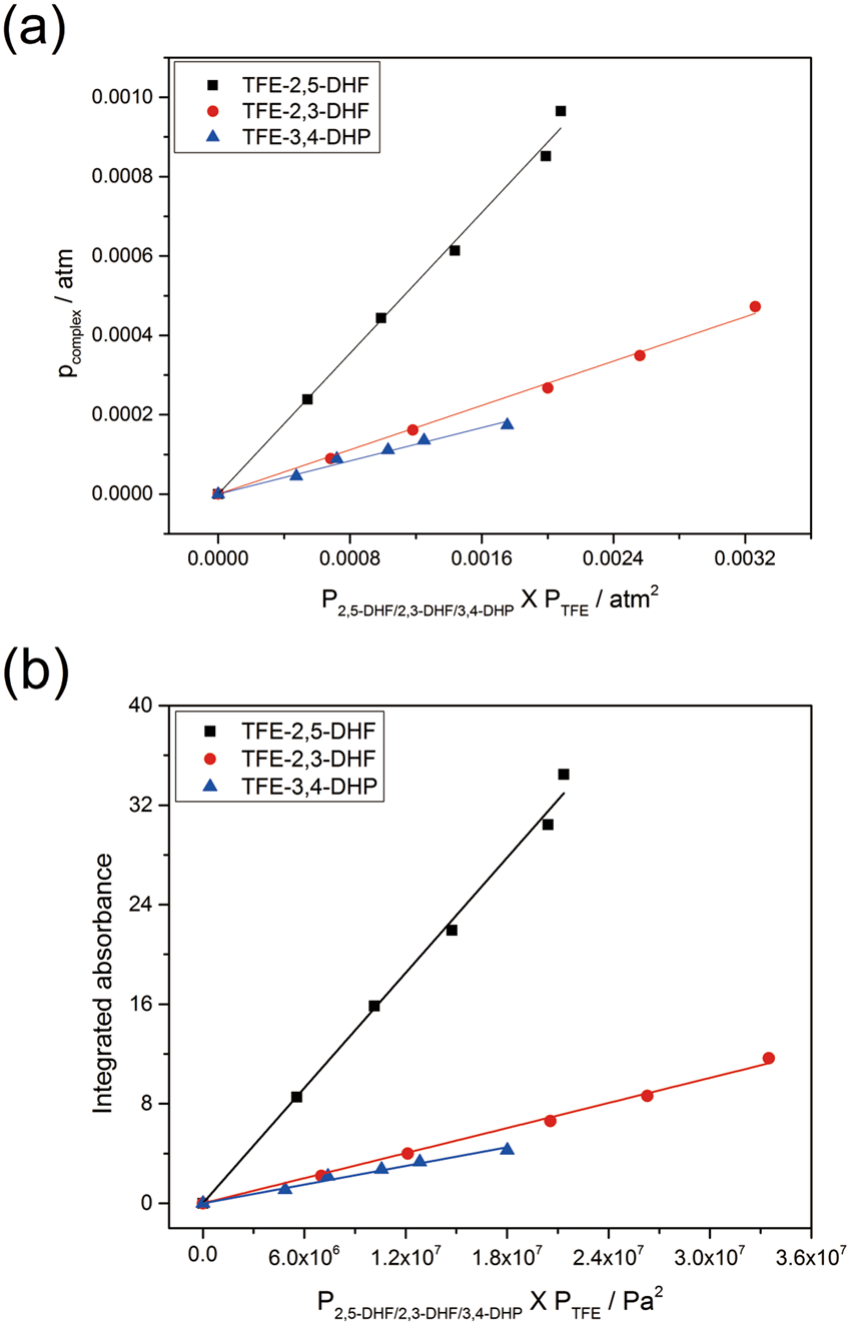
(**a**) Plot of *p*_*complex*_ against *p*_*2,5-DHF/2,3-DHF/3,4-DHP*_ × *p*_*TFE*_; (**b**) The integrated absorbance of the OH-stretching band in the TFE−2,5-DHF, TFE−2,3-DHF and TFE−3,4-DHP complexes as a function of the product of the 2,5-DHF/2,3-DHF/3,4-DHP and TFE pressures. A 20 cm path length cell was used. The integration regions for TFE−2,5-DHF, TFE−2,3-DHF and TFE−3,4-DHP are 3609–3169, 3627–3264 and 3625–3280 cm^−1^, respectively.

The linear correlations between *p*_*complex*_ and the product of the corresponding monomers pressures are shown in Fig. 3(a). The equilibrium constants can be determined by multiplying the slope of the linear fits with the standard pressure according to equation (2). The TFE−2,3-DHF and TFE−3,4-DHP complexes were found to have analogous *K*_*eq*_ values of 0.1397 (297 K) and 0.1047 (299 K), respectively, while the steeper slope of TFE−2,5-DHF has a larger value of 0.4433 (300 K). The highest *K*_*eq*_ value of TFE−2,5-DHF determined, corresponding to the smallest value of Gibbs free energy, suggests that the formation of TFE−2,5-DHF in the gas phase is more favorable and that this complex is stronger than TFE−2,3-DHF and TFE−3,4-DHP. In general, these determined *K*_*eq*_ values for the selected TFE−complexes are comparable with the values obtained from earlier reports on complexes formed between TFE and some other molecules. For example, equilibrium constants of 0.13, 0.3, 0.26 were obtained for TFE−TMP, TFE–ethylene oxide, TFE–dimethylether, respectively^17–19^. However, these *K*_*eq*_ values are about an order of magnitude smaller than those of TFE−TMA (*K*_*eq*_ = 3.5) and TFE−DMA (*K*_*eq*_ = 3.6) complexes^16^, while about an order of magnitude larger when compared with that of TFE−dimethylsulfide (*K*_*eq*_ = 0.052)^18^ complex. The *K*_*eq*_ values listed above suggest moderate thermostability of the complexes formed between TFE and the select heterocyclic compounds in the present study. In addition, all the determined *K*_*eq*_ values of the selected complexes are larger than calculated equilibrium constants listed in Tables 3 and S3, suggesting favorable formation of complexes in experimental conditions than predicted by DFT methods. The integrated absorbance of the OH-stretching band in the TFE−complexes is plotted against the product of the corresponding monomers pressures. A linear dependence can be found for all the complexes as shown in Fig. 3(b), which confirms that the complexes formed are binary. The integration $\int A(\tilde{\nu })d\tilde{\nu }$ is over the entire OH-stretching band as the intensities of the sidebands are really small compared to that of the OH-stretching band. The integration regions for TFE−2,5-DHF, TFE−2,3-DHF and TFE−3,4-DHP are 3169–3609, 3264–3627 and 3280–3625 cm^−1^, respectively.

### AIM analysis

**Figure 4:**
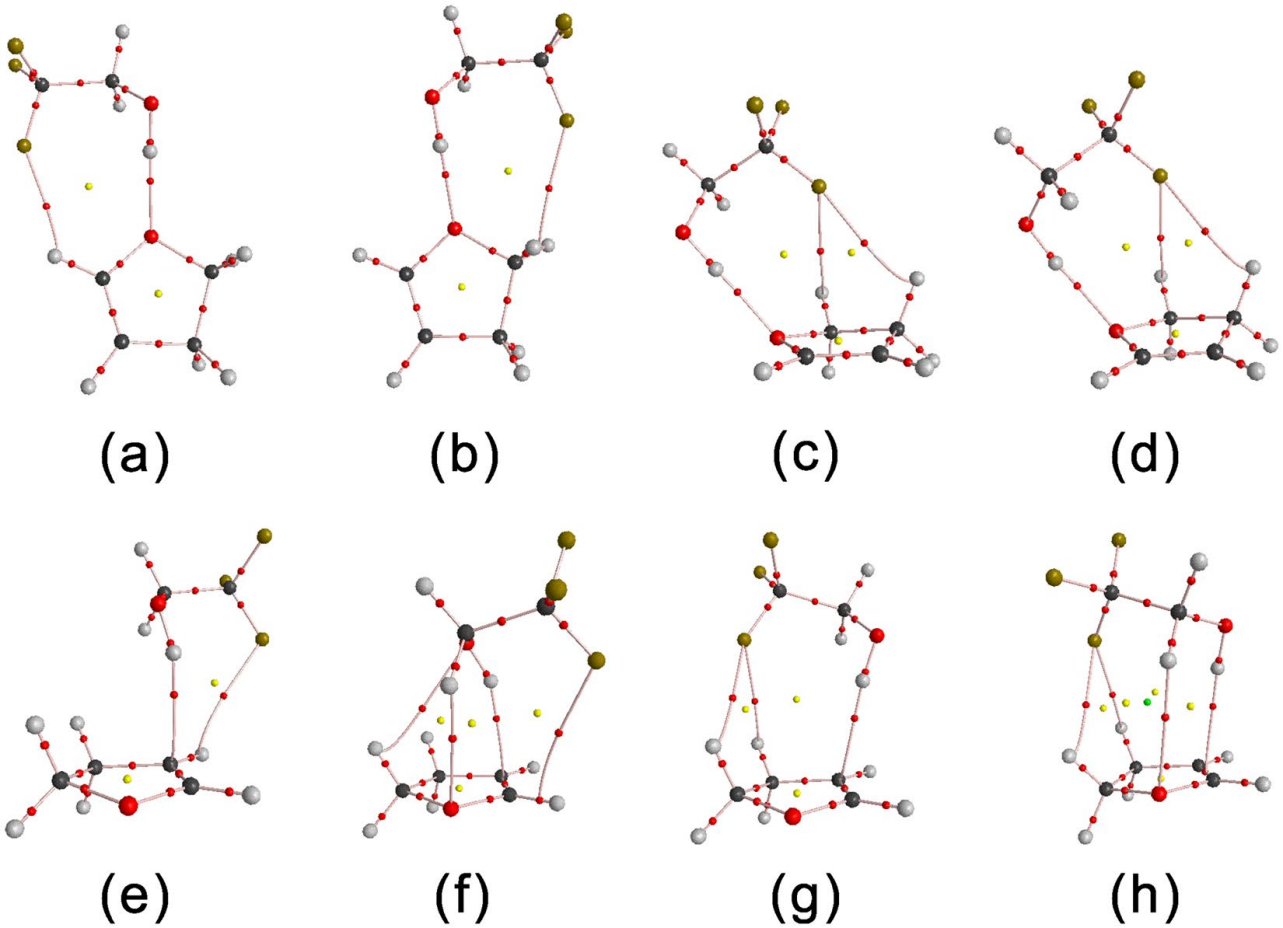
AIM plots of the TFE−2,3-DHF complex obtained with the B3LYP-D3/aug-cc-pVTZ method. The bond critical points, ring critical points and cage critical points are presented by the red, yellow and green balls, respectively.

All the conformers of the TFE−2,3-DHF, TFE−2,5-DHF and TFE−3,4-DHP complexes were analyzed by means of atoms in molecules (AIM) theory. The AIM parameters listed in Table 4 were calculated using the wavefunctions generated at the B3LYP-D3/aug-cc-pVTZ level of theory. AIM parameters obtained at other levels of theory are given in Table S4. The parameters including the change in electronic charge at H atom Δ*q*(H), the change in atomic energy at H atom Δ*E*(H), the electron density *ρ* and Laplacian ∇^2^ *ρ* at the bond critical points (BCPs) for the complexes are listed in Table 4. The correct topology of the gradient vector field is necessary to prove the existence of a hydrogen bond^49^. The AIM plots with (3, −1) BCPs, (3, +1) ring critical points (RCPs) and electron density paths of TFE−2,3-DHF are shown in Fig. 4, while plots of TFE−2,5-DHF and TFE−3,4-DHP are shown in Fig. S4. The formation of hydrogen bonded TFE−complexes can be proved by the presence of BCPs along the line joining the donor and acceptor groups. It can be seen from the plots that in addition to the primary O−H···O or O−H···π hydrogen bonding interactions in the complexes, there also exist other interactions between TFE and the heterocyclic molecules as we can see from the appearance of additional BCPs between the two molecules, which are considered as secondary interactions. These extra interactions are the reason why the hydrogen bond angles deviated from 180° ^34^. From Table 1, we did find that the deviations of O−H···O type hydrogen bonds from linearity are rather small, indicating that interactions other than O−H···O hydrogen bonds are insignificant^6, 43^.

**Table 4:** AIM parameters of the change in electronic charge at H atom Δ*q*(H), the change in atomic energy at H atom Δ*E*(H), the electron density *ρ*(r) and Laplacian ∇^2^ *ρ*(r) at the BCPs for the complexes obtained at the B3LYP-D3/aug-cc-pVTZ level^a^.

Conformer	Type of H-bond	Δ*q*(H)	Δ*E*(H)	*ρ*(BCP)	∇^2^ *ρ*(BCP)
**TFE−2,3-DHF**					
(a)	O−H···O	0.0409	0.0257	0.0348	0.0941
(b)	O−H···O	0.0379	0.0234	0.0350	0.0941
(c)	O−H···O	0.0360	0.0230	0.0334	0.0902
(d)	O−H···O	0.0346	0.0210	0.0332	0.0905
(e)	O−H···π	−0.0034	0.0070	0.0197	0.0407
(f)	O−H···π	−0.0044	0.0042	0.0169	0.0395
(g)	O−H···π	−0.0041	0.0075	0.0186	0.0387
(h)	O−H···π	−0.0053	0.0038	0.0160	0.0376
**TFE−2,5-DHF**					
(a)	O−H···O	0.0454	0.0289	0.0407	0.0984
(b)	O−H···O	0.0439	0.0284	0.0389	0.0960
(c)	O−H···π	−0.0062	0.0029	0.0176	0.0397
(d)	O−H···π	−0.0047	0.0040	0.0171	0.0393
**TFE−3,4-DHP**					
(a)	O−H···O	0.0393	0.0244	0.0348	0.0944
(b)	O−H···O	0.0341	0.0203	0.0350	0.0957
(c)	O−H···O	0.0395	0.0250	0.0336	0.0920
(d)	O−H···O	0.0397	0.0242	0.0336	0.0941
(e)	O−H···π	−0.0025	0.0070	0.0201	0.0420
(f)	O−H···π	−0.0049	0.0043	0.0182	0.0409
(g)	O−H···π	0.0001	0.0103	0.0188	0.0394
(h)	O−H···π	−0.0039	0.0060	0.0177	0.0391

^a^All values are in a.u.

Topological properties at the BCPs can be used to characterize the nature of the hydrogen bonding interactions. The existence of a hydrogen bond can be evidenced by two crucial standards including *ρ* having a value in the range of 0.002 to 0.040 a.u. and ∇^2^ *ρ* having a value that lies between 0.024 and 0.139 a.u. at the BCP^47, 50^. It can be seen from Table 4 that all the values of *ρ* and ∇^2^ *ρ* in the complexes fall well into the corresponding ranges. Thus, the results from AIM analysis also support the formation of the hydrogen bonded TFE−complexes. It has been reported that the value of electron density *ρ* is linearly related to the strength of the hydrogen bond^51, 52^. For the studied complexes, the much lower *ρ* values of O−H···π type conformers relative to those of O−H···O show that the former bond is weaker than the latter. Hence, the dominance of O−H···O type hydrogen bond can be further evidenced from the perspective of electron density. O−H···O type conformers of TFE−2,3-DHF and TFE−3,4-DHP have almost same *ρ* values, suggesting that the hydrogen bonds formed in the two complexes are of similar strength, while the larger *ρ* values of O−H···O type conformers of TFE−2,5-DHF indicate stronger complex formed. In the case of 2,3-DHF and 3,4-DHP rings, the O atom is adjacent to the sp2 carbon. Thus, there is mesomeric effect between the O atom and π-electrons^30, 53, 54^. This effect makes the O atom become deficiency in electrons which further weakens the strength of the O−H···O hydrogen bond. However, this effect doesn’t exist in 2,5-DHF ring as the O atom is far away from the sp2 carbon. Thus, the O atom of 2,5-DHF is richer with electrons compared with those of 2,3-DHF and 3,4-DHP, which is accord with the order of hydrogen bond strengths: TFE−2,5-DHF > TFE−2,3-DHF ≈ TFE−3,4-DHP. Therefore, the directly attachment of the sp2 carbon to the O atom is the essential point for the strength of the O−H···O hydrogen bond. A former AIM analysis of MeOH−2,5-DHF obtained a O−H···O type conformer with electron density of 0.0295 a.u. and a O−H···π type conformer with electron density of 0.0104 a.u.^23^, which are smaller than the corresponding values for TFE−2,5-DHF conformers studied here. This indicates that TFE−2,5-DHF is more stable than MeOH−2,5-DHF due to the electron withdrawing CF_3_ group of TFE.

The larger the change in electronic charge at H atom, Δ*q*(H), the greater the charge transfer from hydrogen bond donor to acceptor. The interaction energy of a hydrogen bond correlates well with the extent of charge transfer^31^. The atomic charges at the H atoms are changed by 0.0439–0.0454, 0.0346–0.0409 and 0.0341–0.0397 a.u. for O−H···O type TFE−2,5-DHF, TFE−2,3-DHF and TFE−3,4-DHP conformers, respectively. These values are quite consistent with characteristics of electron density values, confirming that TFE−2,5-DHF is more stable than TFE−2,3-DHF and TFE−3,4-DHP, which have almost equal stability. In addition, the atomic charges at the H atoms are slightly changed for O−H···π type conformers, which are much smaller than those of corresponding O−H···O type conformers. These values support that O−H···O type conformers of the TFE−complexes are more stable than O−H···π type conformers.

## Conclusions

To study the influence of the position of the double bond and ring size on the stability of hydrogen bonded complexes, the 1:1 complexes formed between TFE and three heterocyclic compounds were identified through FTIR experiments coupled with theoretical calculations. The infrared spectra were measured in the gas phase at room temperature. The recorded spectra were characterized in detail in the region of the OH-stretching fundamental transition. Theoretical calculations demonstrated that all the selected heterocyclic molecules contain two bonding sites available for hydrogen bonding interactions, and the O atom is more energetically favored than π-electrons in all three cases. This is in complete agreement with the calculated harmonic frequencies of O−H···O type isomers being much closer to the experimental observations of OH-stretching fundamental transitions in corresponding complexes. Thus, the observed red shifts in the OH-stretching fundamental transitions were attributed to the formation of O−H···O hydrogen bonded TFE−complexes.

The experimental and theoretical parameters for the complexes vary with the position of the double bond and ring size of the heterocyclic compounds. The OH-stretching fundamental features of TFE−2,3-DHF and TFE−3,4-DHP appear almost at the same position, 3515 and 3510 cm^−1^, respectively, despite changing from five-membered ring to six-membered ring, while when changing the position of the double bond, TFE−2,5-DHF shows a significantly lower frequency of 3458 cm^−1^ relative to that of TFE−2,3-DHF. The following sequence is suggested for the strength of the hydrogen bonds in the complexes: TFE−2,5-DHF > TFE−2,3-DHF ≈ TFE−3,4-DHP. This was further evidenced by the distribution of our determined equilibrium constants and theoretical calculated parameters of vibrations, energetics, geometries and topological analysis. The sequence indicates that the position of the double bond exerts significantly larger influence than ring size on the strength of the selected hydrogen bonded complexes.

## Methods

### Experimental details

TFE (Aladdin, 99.5%), 2,3-DHF (Adamas, 99%), 2,5-DHF (Aladdin, 97%) and 3,4-DHP (Aladdin, 98%) were purified with several freeze, pump and thaw cycles prior to sealing under vacuum. Gas samples of monomers and corresponding mixtures of TFE with each heterocyclic compound were prepared using a 20 cm gas cell connected to a vacuum line with base pressure less than 1 Pa. The gas cell was equipped with CaF_2_ windows. Two Tamagawa CDG-800 pressure gauges connected to the vacuum line were used to measure the pressures of the samples. When preparing the mixtures, known pressure of TFE was filled into the gas cell through the vacuum line, followed by the release of 2,3-DHF/2,5-DHF/3,4-DHP into the gas cell in bursts to allow good mixing. Before measurement of the mixture samples, we allowed monomers to mix for at least 30 minutes to ensure that the infrared spectra were obtained at equilibrium state. Spectra of monomers were recorded at pressures close to corresponding partial pressures used in the mixtures.

The infrared spectra of the samples were recorded on a Bruker Vertex 70 FTIR spectrometer, equipped with KBr beam splitter and a DLaTGS (deuterated lanthanum α alanine doped triglycine sulfate) detector at a resolution of 1 cm^−1^ and 128 scans. Background spectra were recorded with an evacuated cell and subtracted from sample spectra to reduce the interference. The measurements were performed at room temperature (298 ± 2 K). The recorded infrared spectra were then analyzed with OPUS 7.2 program.

### Computational details

Geometry optimizations of TFE, 2,3-DHF, 2,5-DHF and 3,4-DHP monomers and the 1:1 hydrogen bonded TFE−complexes were carried out using the Gaussian 09 program package^55^. All computations were performed with the B3LYP, M06-2X, ωB97X-D and B3LYP-D3 functionals and the aug-cc-pVTZ basis set, using the “opt = verytight” and “int = ultrafine” options^56^. DFT calculations have been used extensively in this field, and the B3LYP-D3 functional has been found to perform well in characterizing the structural, vibrational and thermodynamic properties of complexes^2, 57–59^. This functional was mainly used for the results in this study, while other functionals were used for comparison. The harmonic vibrational frequencies for the optimized geometries were calculated at the same level of theory, and the absence of imaginary frequencies ensured that the optimized structures were minima on the potential surface. The calculated harmonic frequencies are also helpful in the interpretation of infrared spectra.

The *BE*s of the TFE−complexes were determined by subtracting the energy of two monomers from that of complex as^33, 57^:

4 $$BE={E}_{complex}-{E}_{2,5 \mbox{-} DHF/2,3 \mbox{-} DHF/3,4 \mbox{-} DHP}-{E}_{TFE}$$

The calculated *BE*s were corrected by ZPVE and BSSE. BSSE was removed from *BE* using the counterpoise method^44, 60^, and the effect of BSSE on *BE* was found to be minor when a larger basis set was used^33^. Electron density topology analysis using AIM theory has been widely used to analyze the nature of hydrogen bonding interactions, which can provide evidence for the existence of a hydrogen bond^6, 32^. In this work, the topology analysis by means of AIM 2000 program package (version 2) was carried out, the values of electron density and its Laplacian at the bond critical points of hydrogen bonds were used to characterize the hydrogen bonding interactions of the complexes.

### Data Availability

The datasets generated during and/or analyzed during the current study are available from the corresponding author on reasonable request.

## Electronic supplementary material

SUPPLEMENTARY INFO
