# Supplementary material for: The Influence of the Position of the Double Bond and Ring Size on the Stability of Hydrogen Bonded Complexes
Source: Sci Rep. 2017 Sep 12;7:11310. doi: 10.1038/s41598-017-11921-7 (PMC5596019; doi:10.1038/s41598-017-11921-7)
Supplement: Supplementary file 1 — SUPPLEMENTARY INFO [file 41598_2017_11921_MOESM1_ESM.pdf]

Electronic supplementary material

The Influence of the Position of the Double Bond and Ring Size on the Stability of Hydrogen Bonded Complexes

Shumin Cheng, Shanshan Tang, Narcisse T. Tsona, Lin Du*

Environment Research Institute, Shandong University, Shanda South Road 27, 250100 Shandong, China

e-mail: lindu@sdu.edu.cn

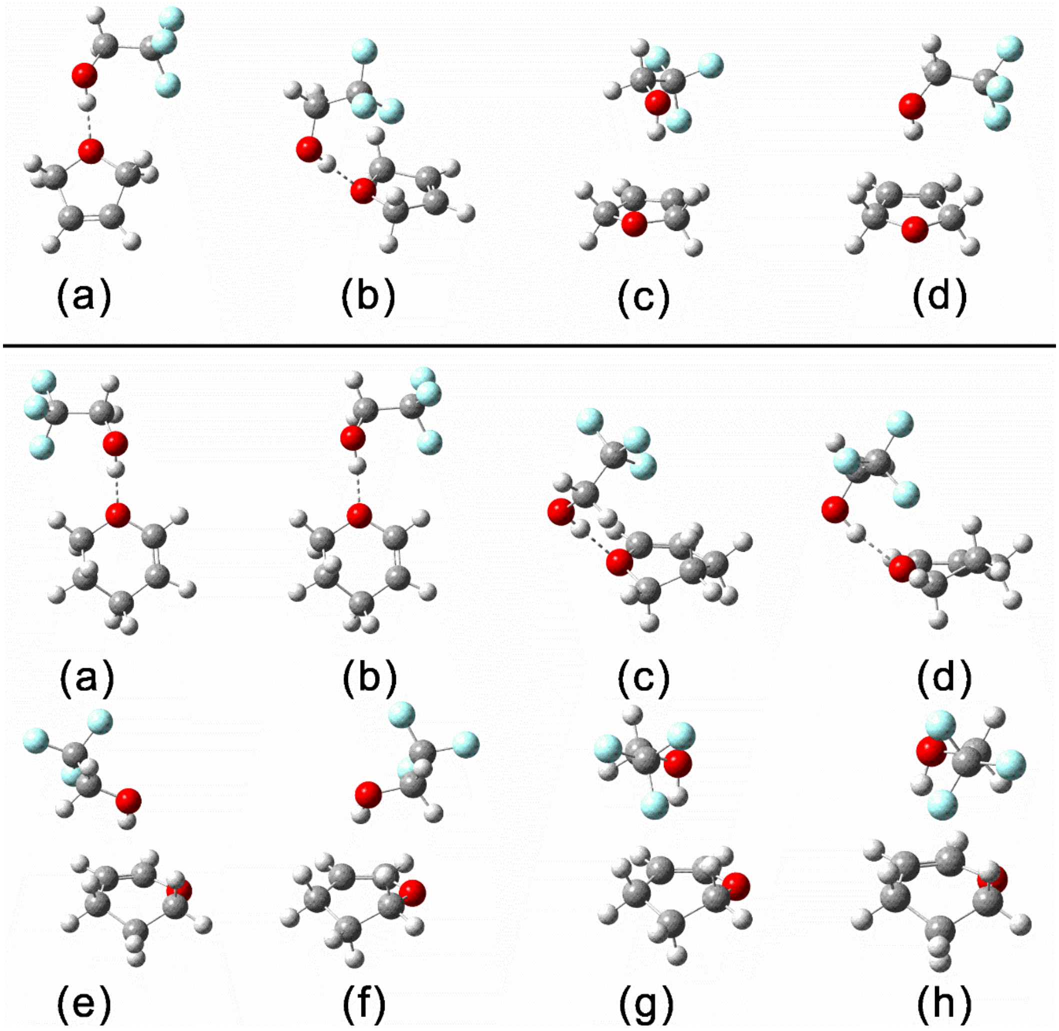

Figure S1. Optimized structures of the TFE−2,5-DHF (top) and TFE−3,4-DHP (bottom) complexes calculated at the B3LYP-D3/aug-cc-pVTZ level of theory.

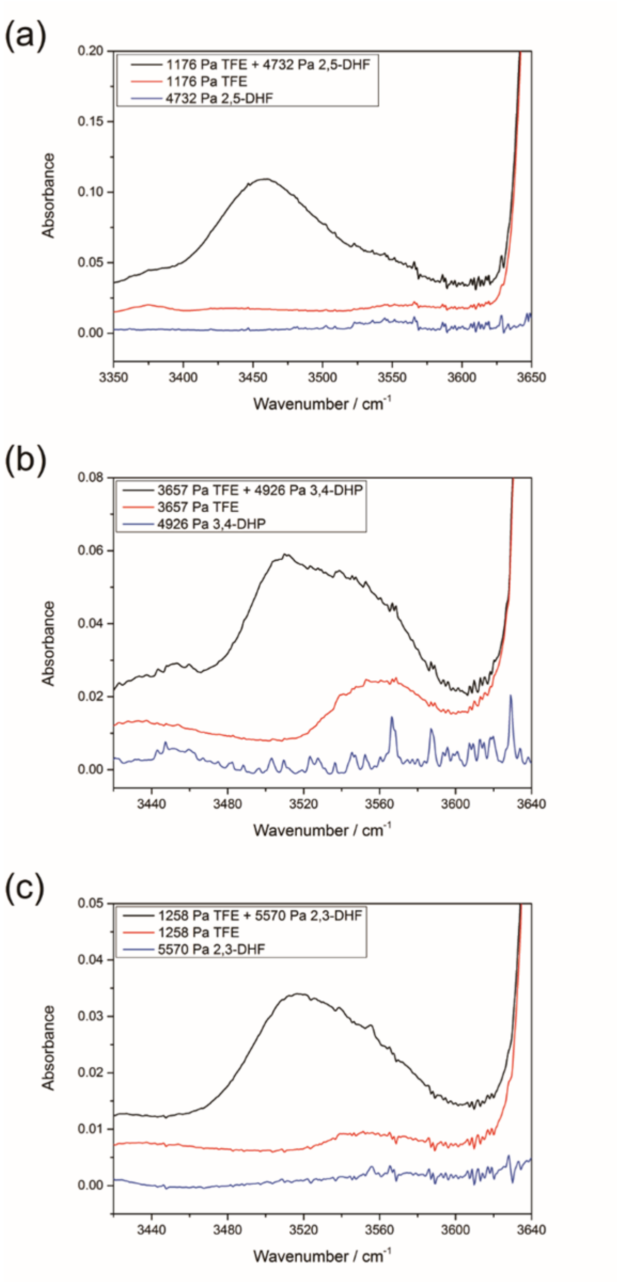

Figure S2. (a) Spectra of TFE, 2,5-DHF, and their mixture in the 3350-3650 cm^{-1} region. (b) Spectra of TFE, 3,4-DHP, and their mixture in the 3420-3640 cm^{-1} region. (c) Spectra of TFE, 2,3-DHF, and their mixture in the 3420-3640 cm^{-1} region. A 20 cm path length cell was used.

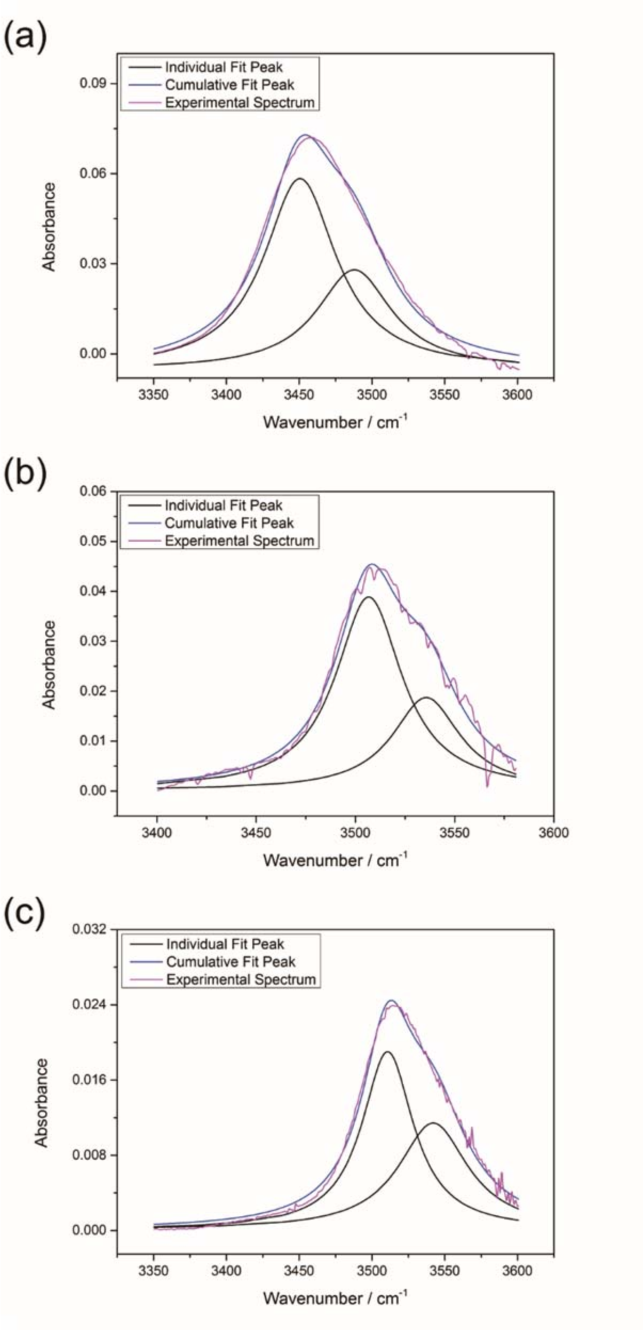

Figure S3. The deconvolution fittings of the OH-stretching fundamental transition bands of TFE−2,5-DHF (a), TFE−3,4-DHP (b) and TFE−2,3-DHF (c).

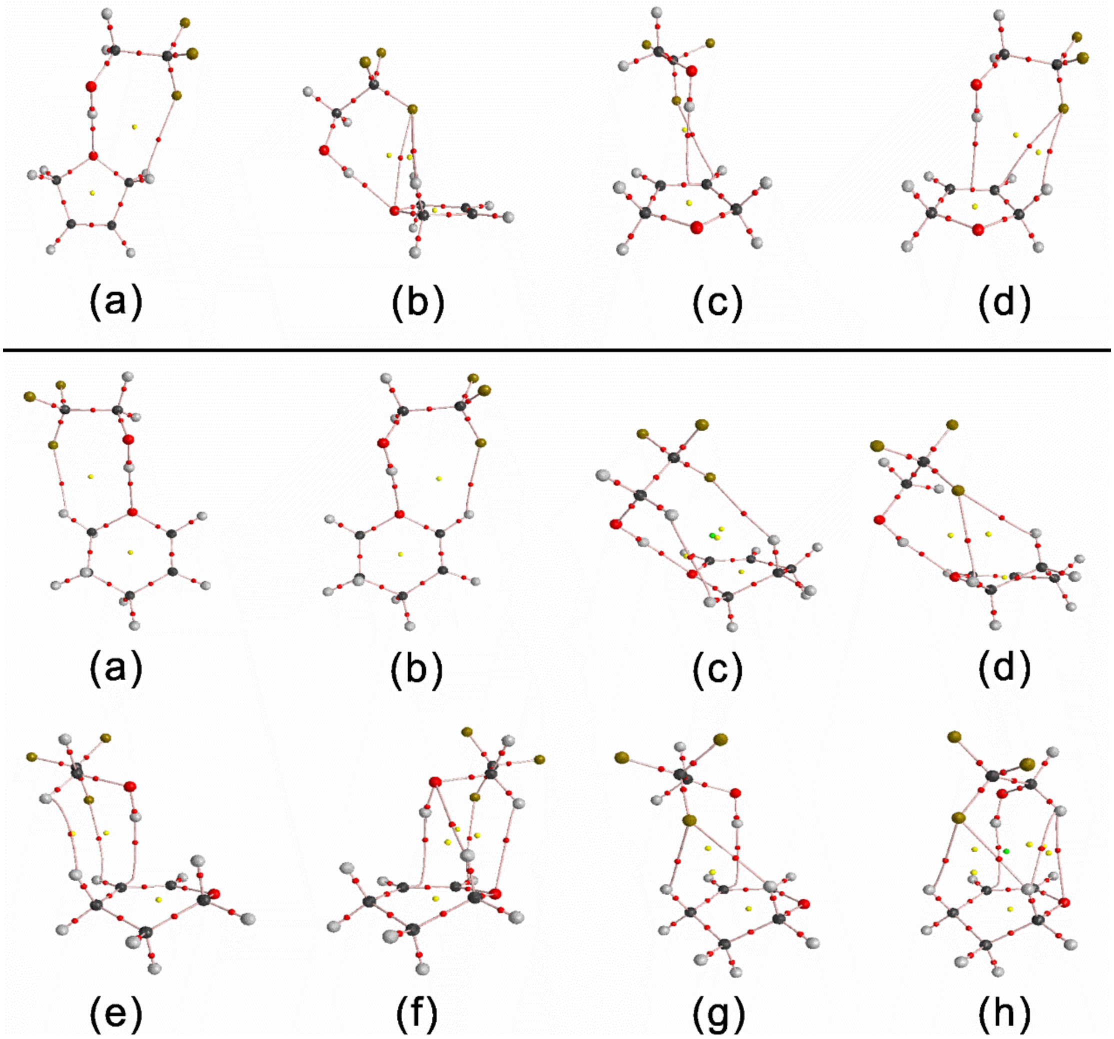

Figure S4. AIM plots of TFE−2,5-DHF (top) and TFE−3,4-DHP (bottom) complexes obtained with the B3LYP-D3/aug-cc-pVTZ method. The bond critical points, ring critical points and cage critical points are presented by the red, yellow and green balls, respectively.

Table S1. Selected optimized geometric parameters in the complexes (angles in degrees and bond lengths in Å)

Conformer	Type of H-bond	Method [a]	$r_{(OH)}$ [b]	$\Delta r_{(OH)}$ [c]	$r_{(HB)}$ [d]	$\theta_{(HB)}$ [e]
TFE−2,3-DHF						
(a)	O−H···O	B3LYP	0.9726	0.0097	1.8589	175.7
		M06-2X	0.9693	0.0078	1.8522	165.3
		ωB97X-D	0.9688	0.0104	1.8232	174.4
(b)	O−H···O	B3LYP	0.9726	0.0097	1.8564	176.6
		M06-2X	0.9696	0.0082	1.8376	170.9
		ωB97X-D	0.9687	0.0103	1.8213	177.1
(c)[f]	O−H···O	B3LYP	-	-	-	-
		M06-2X	0.9710	0.0095	1.8587	161.8
		ωB97X-D	0.9693	0.0109	1.8382	177.2
(d)[f]	O−H···O	B3LYP	-	-	-	-
		M06-2X	0.9699	0.0084	1.8627	166.2
		ωB97X-D	0.9682	0.0098	1.8534	169.1
(e)	O−H···π	B3LYP	0.9707	0.0078	-	-
		M06-2X	0.9676	0.0062	-	-
		ωB97X-D	0.9663	0.0079	-	-
(f)	O−H···π	B3LYP	0.9705	0.0076	-	-
		M06-2X	0.9670	0.0055	-	-
		ωB97X-D	0.9652	0.0068	-	-
(g)	O−H···π	B3LYP	0.9700	0.0071	-	-
		M06-2X	0.9667	0.0052	-	-
		ωB97X-D	0.9657	0.0073	-	-
(h)	O−H···π	B3LYP	0.9699	0.0070	-	-
		M06-2X	0.9654	0.0040	-	-
		ωB97X-D	0.9642	0.0058	-	-
TFE−2,5-DHF						
(a)	O−H···O	B3LYP	0.9763	0.0134	1.8013	176.3
		M06-2X	0.9750	0.0135	1.7599	177.8
		ωB97X-D	0.9730	0.0146	1.7637	178.0
(b)[f]	O−H···O	B3LYP	-	-	-	-
		M06-2X	0.9748	0.0133	1.7876	168.9
		ωB97X-D	0.9728	0.0144	1.7827	173.2
(c)[f]	O−H···π	B3LYP	-	-	-	-
		M06-2X	0.9657	0.0042	-	-

		ωB97X-D	0.9641	0.0057	-	-
(d)	O−H···π	B3LYP	0.9657	0.0028	-	-
		M06-2X	0.9650	0.0035	-	-
		ωB97X-D	0.9636	0.0052	-	-
TFE−3,4-DHP						
(a)	O−H···O	B3LYP	0.9725	0.0096	1.8592	175.1
		M06-2X	0.9679	0.0064	1.9236	148.5
		ωB97X-D	0.9680	0.0096	1.8385	165.7
(b)	O−H···O	B3LYP	0.9726	0.0098	1.8559	172.6
		M06-2X	0.9691	0.0076	1.8552	159.5
		ωB97X-D	0.9687	0.0103	1.8215	170.1
(c)[f]	O−H···O	B3LYP	-	-	-	-
		M06-2X	0.9698	0.0083	1.8654	161.7
		ωB97X-D	0.9685	0.0101	1.8486	167.7
(d)[f]	O−H···O	B3LYP	-	-	-	-
		M06-2X	0.9675	0.0060	1.9386	149.4
		ωB97X-D	0.9666	0.0082	1.8881	158.5
(e)	O−H···π	B3LYP	0.9707	0.0078	-	-
		M06-2X	0.9681	0.0066	-	-
		ωB97X-D	0.9666	0.0082	-	-
(f)	O−H···π	B3LYP	0.9706	0.0077	-	-
		M06-2X	0.9676	0.0061	-	-
		ωB97X-D	0.9659	0.0075	-	-
(g)	O−H···π	B3LYP	0.9698	0.0069	-	-
		M06-2X	0.9666	0.0051	-	-
		ωB97X-D	0.9657	0.0073	-	-
(h)	O−H···π	B3LYP	0.9697	0.0068	-	-
		M06-2X	0.9656	0.0041	-	-
		ωB97X-D	0.9648	0.0064	-	-

[a] Using the aug-cc-pVTZ basis set. [b] OH bond length. [c] $\Delta r_{(OH)} = r_{complex} - r_{TFE}$, is the change in the OH bond length upon complexation. [d] Intermolecular hydrogen bond distance. [e] Intermolecular hydrogen bond angle, i.e., $\theta_{(O-H\cdots O)}$. [f] Not obtained at the B3LYP/aug-cc-pVTZ level.

Table S2. Calculated harmonic OH-stretching wavenumbers (in cm^{-1}) and oscillator strengths [a]

Conformer	Type of H-bond		B3LYP	M06-2X	ωB97X-D
TFE		$\tilde{\nu}$	3806	3869	3892
		f_{TFE}	9.2×10^{-6}	1.2×10^{-5}	9.5×10^{-6}
TFE−2,3-DHF					
(a)	O−H···O	$\tilde{\nu}$	3603	3704	3682
		$\Delta\tilde{\nu}$ [b]	202	165	209
		f	1.6×10^{-4}	1.4×10^{-4}	1.6×10^{-4}
		f/f_{TFE}	17.5	11.4	16.8
(b)	O−H···O	$\tilde{\nu}$	3605	3692	3683
		$\Delta\tilde{\nu}$ [b]	201	177	208
		f	1.6×10^{-4}	1.5×10^{-4}	1.6×10^{-4}
		f/f_{TFE}	17.4	12.2	16.8
(c)[c]	O−H···O	$\tilde{\nu}$	-	3670	3671
		$\Delta\tilde{\nu}$ [b]	-	199	221
		f	-	1.2×10^{-4}	1.4×10^{-4}
		f/f_{TFE}	-	9.8	14.5
(d)[c]	O−H···O	$\tilde{\nu}$	-	3688	3687
		$\Delta\tilde{\nu}$ [b]	-	181	205
		f	-	1.1×10^{-4}	1.2×10^{-4}
		f/f_{TFE}	-	8.9	12.0
(e)	O−H···π	$\tilde{\nu}$	3634	3745	3726
		$\Delta\tilde{\nu}$ [b]	171.5	124.5	166
		f	1.0×10^{-4}	6.1×10^{-5}	7.9×10^{-5}
		f/f_{TFE}	11.0	4.9	8.3
(f)	O−H···π	$\tilde{\nu}$	3638	3766	3748
		$\Delta\tilde{\nu}$ [b]	167	103	143
		f	9.6×10^{-5}	4.9×10^{-5}	5.9×10^{-5}
		f/f_{TFE}	10.5	4.0	6.2
(g)	O−H···π	$\tilde{\nu}$	3649	3764	3733
		$\Delta\tilde{\nu}$ [b]	157	105	158
		f	9.7×10^{-5}	6.6×10^{-5}	9.2×10^{-5}
		f/f_{TFE}	10.6	5.3	9.6
(h)	O−H···π	$\tilde{\nu}$	3650	3789	3765
		$\Delta\tilde{\nu}$ [b]	156	80	127
		f	9.5×10^{-5}	4.6×10^{-5}	6.5×10^{-5}
		f/f_{TFE}	10.4	3.7	6.9
TFE−2,5-DHF					
(a)	O−H···O	$\tilde{\nu}$	3528	3555	3586
		$\Delta\tilde{\nu}$ [b]	278	314	305
		f	1.8×10^{-4}	1.9×10^{-4}	1.8×10^{-4}
		f/f_{TFE}	19.6	15.3	18.7
(b)[c]	O−H···O	$\tilde{\nu}$	-	3572	3586
		$\Delta\tilde{\nu}$ [b]	-	297	305
		f	-	1.5×10^{-4}	1.5×10^{-4}
		f/f_{TFE}	-	12.2	16.2

(c)[c]	O−H···π	$\tilde{\nu}$	-	3783	3768
		$\Delta\tilde{\nu}^{b}$	-	86	123
		f	-	5.9×10^{-5}	7.4×10^{-5}
		f/f_{TFE}	-	4.8	7.7
(d)	O−H···π	$\tilde{\nu}$	3738	3800	3779
		$\Delta\tilde{\nu}^{b}$	68	69	112
		f	4.3×10^{-5}	5.0×10^{-5}	7.0×10^{-5}
		f/f_{TFE}	4.7	4.1	7.3
TFE−3,4-DHP					
(a)	O−H···O	$\tilde{\nu}$	3605	3751	3697
		$\Delta\tilde{\nu}^{b}$	201	118	708
		f	1.6×10^{-4}	7.1×10^{-5}	1.3×10^{-4}
		f/f_{TFE}	17.5	5.7	13.9
(b)	O−H···O	$\tilde{\nu}$	3603	3710	3686
		$\Delta\tilde{\nu}^{b}$	203	159	206
		f	1.6×10^{-4}	1.3×10^{-4}	1.6×10^{-4}
		f/f_{TFE}	17.4	10.5	16.6
(c)[c]	O−H···O	$\tilde{\nu}$	-	3699	3683
		$\Delta\tilde{\nu}^{b}$	-	170	208
		f	-	1.0×10^{-4}	1.2×10^{-4}
		f/f_{TFE}	-	8.4	12.4
(d)[c]	O−H···O	$\tilde{\nu}$	-	3759	3726
		$\Delta\tilde{\nu}^{b}$	-	110	165
		f	-	6.1×10^{-5}	8.7×10^{-5}
		f/f_{TFE}	-	4.9	9.2
(e)	O−H···π	$\tilde{\nu}$	3636	3739	3719
		$\Delta\tilde{\nu}^{b}$	170	130	172
		f	9.7×10^{-5}	6.9×10^{-5}	8.3×10^{-5}
		f/f_{TFE}	10.6	5.6	8.7
(f)	O−H···π	$\tilde{\nu}$	3636	3745	3732
		$\Delta\tilde{\nu}^{b}$	170	124	160
		f	9.7×10^{-5}	5.9×10^{-5}	6.7×10^{-5}
		f/f_{TFE}	10.6	4.7	7.0
(g)	O−H···π	$\tilde{\nu}$	3655	3763	3730.6
		$\Delta\tilde{\nu}^{b}$	151	106	161.0
		f	9.5×10^{-5}	7.0×10^{-5}	9.6×10^{-5}
		f/f_{TFE}	10.4	5.6	10.1
(h)	O−H···π	$\tilde{\nu}$	3654.3	3783	3753
		$\Delta\tilde{\nu}^{b}$	151.6	85.7	138.3
		f	9.5×10^{-5}	5.2×10^{-5}	7.5×10^{-5}
		f/f_{TFE}	10.4	4.2	7.8

[a] Using the aug-cc-pVTZ basis set. [b] $\Delta\tilde{\nu}_{OH} = \tilde{\nu}_{TFE} - \tilde{\nu}_{complex}$. [c] Not obtained at the B3LYP/aug-cc-pVTZ level.

Table S3. Calculated binding energy (*BE*), enthalpy of formation (ΔH^{θ}_{calc}), Gibbs free energy of formation (ΔG^{θ}_{calc}) and equilibrium constant (K^{calc}_{eq}) at 298 K for the complexes

Conformer	Type of H-bond	Method [a]	*BE* [b]	ZPVE	BSSE	ΔH^{θ}_{calc}	ΔG^{θ}_{calc}	K^{calc}_{eq}
TFE−2,3-DHF								
(a)	O−H···O	B3LYP	-18.9	3.9	0.9	-17.5	16.5	1.3×10^{-3}
		M06-2X	-25.5	3.7	1.0	-24.1	10.0	1.8×10^{-2}
		ωB97X-D	-25.4	4.2	1.1	-24.3	8.7	3.0×10^{-2}
(b)	O−H···O	B3LYP	-18.9	3.9	0.8	-17.4	16.0	1.5×10^{-3}
		M06-2X	-25.9	3.9	1.1	-24.7	8.8	2.9×10^{-2}
		ωB97X-D	-25.4	4.5	1.2	-24.5	10.6	1.4×10^{-2}
(c)[c]	O−H···O	B3LYP	-	-	-	-	-	-
		M06-2X	-25.2	4.0	1.6	-24.9	13.1	5.0×10^{-3}
		ωB97X-D	-24.3	4.9	1.4	-24.3	16.4	1.3×10^{-3}
(d)[c]	O−H···O	B3LYP	-	-	-	-	-	-
		M06-2X	-27.2	4.8	1.5	-27.3	14.0	3.5×10^{-3}
		ωB97X-D	-26.9	4.9	1.4	-26.8	13.5	4.3×10^{-3}
(e)	O−H···π	B3LYP	-8.4	2.8	0.8	-6.6	24.9	4.3×10^{-5}
		M06-2X	-18.8	3.2	1.1	-17.8	19.8	3.4×10^{-4}
		ωB97X-D	-19.7	3.5	1.1	-18.6	18.1	6.8×10^{-4}
(f)	O−H···π	B3LYP	-9.6	2.7	0.7	-7.6	22.1	1.3×10^{-4}
		M06-2X	-23.2	4.0	1.4	-22.7	17.3	9.1×10^{-4}
		ωB97X-D	-22.7	4.1	1.2	-22.0	17.5	8.5×10^{-4}
(g)	O−H···π	B3LYP	-8.1	3.0	0.8	-6.5	27.1	1.7×10^{-5}
		M06-2X	-17.9	3.1	1.5	-17.0	19.0	4.6×10^{-4}
		ωB97X-D	-19.2	3.7	1.3	-18.4	18.8	5.1×10^{-4}
(h)	O−H···π	B3LYP	-9.5	3.0	0.9	-7.8	26.4	2.4×10^{-5}
		M06-2X	-21.4	3.9	1.6	-21.2	20.9	2.2×10^{-4}
		ωB97X-D	-21.5	4.6	1.5	-21.4	20.8	2.3×10^{-4}
TFE−2,5-DHF								
(a)	O−H···O	B3LYP	-23.3	4.6	0.8	-22.1	12.5	6.6×10^{-3}
		M06-2X	-30.7	4.5	1.2	-30.0	6.1	8.5×10^{-2}
		ωB97X-D	-30.9	5.0	1.3	-30.4	6.0	8.9×10^{-2}
(b)[c]	O−H···O	B3LYP	-	-	-	-	-	-
		M06-2X	-31.3	4.9	1.7	-31.8	10.3	1.5×10^{-2}
		ωB97X-D	-30.5	5.6	1.6	-31.1	11.0	1.2×10^{-2}
(c)[c]	O−H···π	B3LYP	-	-	-	-	-	-
		M06-2X	-14.5	2.9	1.2	-13.3	23.5	7.6×10^{-5}

		ωB97X-D	-16.3	2.5	1.2	-14.7	16.3	1.4×10^{-3}
		B3LYP	-5.5	3.2	0.9	-3.9	32.1	2.4×10^{-6}
(d)	O−H···π	M06-2X	-13.7	3.8	1.5	-13.5	28.6	9.7×10^{-6}
		ωB97X-D	-14.8	3.3	1.3	-13.9	22.8	9.9×10^{-5}
TFE−3,4-DHP								
		B3LYP	-18.5	3.8	0.9	-16.9	16.3	1.4×10^{-3}
(a)	O−H···O	M06-2X	-26.1	4.0	1.3	-25.4	13.0	5.3×10^{-3}
		ωB97X-D	-25.9	4.1	1.2	-24.9	11.1	1.1×10^{-2}
		B3LYP	-18.3	3.8	0.8	-16.6	16.8	1.1×10^{-3}
(b)	O−H···O	M06-2X	-25.4	3.5	1.0	-23.9	9.3	2.3×10^{-2}
		ωB97X-D	-25.5	4.0	1.1	-24.3	8.7	3.0×10^{-2}
		B3LYP	-	-	-	-	-	-
(c)[c]	O−H···O	M06-2X	-24.1	4.0	1.5	-23.7	16.0	1.6×10^{-3}
		ωB97X-D	-25.3	4.2	1.5	-25.0	15.4	2.0×10^{-3}
		B3LYP	-	-	-	-	-	-
(d)[c]	O−H···O	M06-2X	-28.1	4.6	1.6	-28.1	14.8	2.6×10^{-3}
		ωB97X-D	-28.0	4.5	1.6	-27.8	12.7	5.8×10^{-3}
		B3LYP	-9.0	2.7	0.8	-6.9	24.1	6.1×10^{-5}
(e)	O−H···π	M06-2X	-19.9	3.5	1.2	-18.9	19.9	3.3×10^{-4}
		ωB97X-D	-21.4	3.6	1.2	-20.4	16.7	1.2×10^{-3}
		B3LYP	-10.1	2.7	0.8	-8.0	22.2	1.3×10^{-4}
(f)	O−H···π	M06-2X	-23.2	3.9	1.4.	-22.6	17.4	8.8×10^{-4}
		ωB97X-D	-24.1	4.0	1.3	-23.4	16.4	1.3×10^{-3}
		B3LYP	-8.5	2.7	0.9	-6.6	26.8	2.0×10^{-5}
(g)	O−H···π	M06-2X	-18.7	3.0	1.5	-17.6	19.6	3.7×10^{-4}
		ωB97X-D	-20.9	3.5	1.5	-20.2	18.0	6.9×10^{-4}
		B3LYP	-9.7	2.7	0.9	-7.8	25.3	3.7×10^{-5}
(h)	O−H···π	M06-2X	-21.5	3.6	1.6	-21.0	20.1	3.0×10^{-4}
		ωB97X-D	-23.5	3.4	1.6	-22.7	15.6	1.8×10^{-3}

[a] Using the aug-cc-pVTZ basis set. Energies are in kJ mol^{-1}. [b] *BE* corrected with ZPVE and BSSE.

[c] Not obtained at the B3LYP/aug-cc-pVTZ level.

Table S4. AIM parameters for the complexes [a]

Conformer		Type of H-bond	Δq(H)	ΔE(H)	ρ(BCP)	$\nabla^2\rho$(BCP)
B3LYP						
TFE−2,3-DHF	(a)	O−H···O	0.0352	0.0221	0.0308	0.0893
	(b)	O−H···O	0.0352	0.0217	0.0309	0.0898
	(c)[b]	O−H···O	-	-	-	-
	(d)[b]	O−H···O	-	-	-	-
	(e)	O−H···π	-0.0043	0.0079	0.0162	0.0351
	(f)	O−H···π	-0.0069	0.0054	0.0162	0.0352
	(g)	O−H···π	-0.0054	0.0070	0.0149	0.0334
	(h)	O−H···π	-0.0080	0.0043	0.0154	0.0343
TFE−2,5-DHF	(a)	O−H···O	0.0440	0.0286	0.0362	0.0945
	(b)[b]	O−H···O	-	-	-	-
	(c)[b]	O−H···π	-	-	-	-
	(d)	O−H···π	-0.0064	0.0015	0.0106	0.0277
TFE−3,4-DHP	(a)	O−H···O	0.0362	0.0229	0.0308	0.0892
	(b)	O−H···O	0.0385	0.0246	0.0310	0.0901
	(c)[b]	O−H···O	-	-	-	-
	(d)[b]	O−H···O	-	-	-	-
	(e)	O−H···π	-0.0051	0.0066	0.0161	0.0357
	(f)	O−H···π	-0.0056	0.0064	0.0165	0.0362
	(g)	O−H···π	-0.0023	0.0089	0.0145	0.0334
	(h)	O−H···π	-0.0090	0.0032	0.0150	0.0344
M06-2X						
TFE−2,3-DHF	(a)	O−H···O	0.0482	0.0301	0.0301	0.1031
	(b)	O−H···O	0.0492	0.0304	0.0310	0.1039
	(c)	O−H···O	0.0437	0.0280	0.0300	0.1009
	(d)	O−H···O	0.0477	0.0300	0.0301	0.0998
	(e)	O−H···π	0.0058	0.0103	0.0170	0.0448
	(f)	O−H···π	0.0061	0.0092	0.0154	0.0448
	(g)	O−H···π	0.0048	0.0105	0.0152	0.0404
	(h)	O−H···π	0.0069	0.0118	0.0139	0.0383
TFE−2,5-DHF	(a)	O−H···O	0.0530	0.0330	0.0393	0.1087
	(b)	O−H···O	0.0527	0.0335	0.0365	0.1069
	(c)	O−H···π	0.0082	0.0110	0.0162	0.0440
	(d)	O−H···π	0.0057	0.0094	0.0141	0.0399
TFE−3,4-DHP	(a)	O−H···O	0.0374	0.0223	0.0262	0.0994
	(b)	O−H···O	0.0427	0.0253	0.0300	0.1051
	(c)	O−H···O	0.0465	0.0286	0.0294	0.1019
	(d)	O−H···O	0.0379	0.0231	0.0252	0.0960
	(e)	O−H···π	0.0104	0.0145	0.0178	0.0459
	(f)	O−H···π	0.0038	0.0090	0.0173	0.0454
	(g)	O−H···π	0.0065	0.0115	0.0154	0.0411
	(h)	O−H···π	0.0061	0.0107	0.0144	0.0396
ωB97X-D						
TFE−2,3-DHF	(a)	O−H···O	0.0409	0.0283	0.0337	0.0943
	(b)	O−H···O	0.0399	0.0274	0.0338	0.0945
	(c)	O−H···O	0.0396	0.0281	0.0328	0.0909
	(d)	O−H···O	0.0403	0.0284	0.0320	0.0902

	(e)	O−H···π	0.0056	0.0153	0.0188	0.0410
	(f)	O−H···π	0.0053	0.0140	0.0172	0.0399
	(g)	O−H···π	0.0080	0.0179	0.0181	0.0394
	(h)	O−H···π	0.0048	0.0143	0.0163	0.0381
TFE−2,5-DHF	(a)	O−H···O	0.0519	0.0369	0.0400	0.0990
	(b)	O−H···O	0.0471	0.0333	0.0381	0.0970
	(c)	O−H···π	0.0058	0.0139	0.0173	0.0400
	(d)	O−H···π	0.0037	0.0127	0.0163	0.0395
TFE−3,4-DHP	(a)	O−H···O	0.0419	0.0288	0.0326	0.0948
	(b)	O−H···O	0.0444	0.0314	0.0337	0.0956
	(c)	O−H···O	0.0419	0.0295	0.0320	0.0918
	(d)	O−H···O	0.0351	0.0236	0.0294	0.0903
	(e)	O−H···π	0.0040	0.0139	0.0194	0.0421
	(f)	O−H···π	0.0006	0.0106	0.0183	0.0412
	(g)	O−H···π	0.0091	0.0185	0.0186	0.0402
	(h)	O−H···π	0.0037	0.0133	0.0174	0.0396

[a] Using the aug-cc-pVTZ basis set. All values are in a.u. [b] Not obtained at the B3LYP/aug-cc-pVTZ level.
